# Inhibitory effect of traditional oriental medicine-derived monoamine oxidase B inhibitor on radioresistance of non-small cell lung cancer

**DOI:** 10.1038/srep21986

**Published:** 2016-02-24

**Authors:** Beomseok Son, Se Young Jun, HyunJeong Seo, HyeSook Youn, Hee Jung Yang, Wanyeon Kim, Hyung Kook Kim, ChulHee Kang, BuHyun Youn

**Affiliations:** 1 Department of Integrated Biological Science, Pusan National University, Busan, Republic of Korea; 2 Department of Chemistry, Washington State University, Pullman, Washington, USA; 3 Nuclear Science Research Institute, Pusan National University, Busan, Republic of Korea; 4 Department of Biological Sciences, Pusan National University, Busan, Republic of Korea; 5 Department of Nanomaterial Engineering and Nanoconvergence Technology, Pusan National University, Miryang, Republic of Korea

## Abstract

Increased survival of cancer cells mediated by high levels of ionizing radiation (IR) reduces the effectiveness of radiation therapy for non-small cell lung cancer (NSCLC). In the present study, danshensu which is a selected component of traditional oriental medicine (TOM) compound was found to reduce the radioresistance of NSCLC by inhibiting the nuclear factor-κB (NF-κB) pathway. Of the various TOM compounds reported to inhibit the IR activation of NF-κB, danshensu was chosen as a final candidate based on the results of structural comparisons with human metabolites and monoamine oxidase B (MAOB) was identified as the putative target enzyme. Danshensu decreased the activation of NF-κB by inhibiting MAOB activity in A549 and NCI-H1299 NSCLC cells. Moreover, it suppressed IR-induced epithelial-to-mesenchymal transition, expressions of NF-κB-regulated prosurvival and proinflammatory genes, and *in vivo* radioresistance of mouse xenograft models. Taken together, this study shows that danshensu significantly reduces MAOB activity and attenuates NF-κB signaling to elicit the radiosensitization of NSCLC.

A large proportion of cancer-related deaths are caused by lung cancer, which remains one of the most common cancers worldwide. Furthermore, many patients are not diagnosed until the disease has reached an advanced stage. Non-small cell lung cancer (NSCLC) accounts for more than 85% of lung cancer cases and the improvements made in its treatment over the last few decades have been less than satisfactory^1^. Radiation therapy can be used to treat tumors including inoperable NSCLC by inducing cancer-specific damage^2^. Nevertheless, NSCLC cells acquire radioresistance, which overwhelms the benefit of therapy and substantially increases mortality^3^. In most cases, distant metastasis or secondary lung cancer develops following the failure of radiotherapy, and it has been reported that the occurrence of remote metastases is well correlated with overall mortality in early-phase NSCLC^4^. A sequence of events that include epithelial-to-mesenchymal transition (EMT) are required for cancer metastasis to take place^5^, which indicates that the development of potent adjuvants that limit radiation-induced EMT and radioresistance in NSCLC cells is needed to prevent metastasis and enhance therapeutic outcomes.

Previous findings suggest that ionizing radiation (IR) is able to affect the expressions and activities of transcription factors and alter a variety of signaling pathways mediating EMT and radioresistance^6^. In particular, nuclear factor-κB (NF-κB) signaling constitutes a key cellular signal transduction pathway that is involved in innate and adaptive immunity, inflammation, stress responses, and proliferation^7^. When cells are irradiated with low or high doses of IR, the damage induces increases of NF-κB expression and consequently the expressions of anti-apoptotic genes, such as *BCL2*, *BCL2L1*, *MCL1*, and *CFLA*^8^. In fact, the constitutive activation of NF-κB is relevant in the context of tumorigenesis, resistance to chemo/radiotherapy, and metastasis. Thus, it is not surprising that its activation is regarded as a marker of tumor radioresistance and metastasis. NF-κB inhibitors have been used to improve the efficacies of anticancer treatments and radiotherapy^9^,^10^. In addition, a number of anti-inflammatory natural compounds, such as triptolide, celastrol, evodiamine, and wogonin are known to inhibit the NF-κB signaling cascade^11^.

Traditional oriental medicine (TOM) has been used clinically for thousands of years and the natural products contained in TOM sometimes provide valuable insights regarding the developments of drugs for modern medicine, as exemplified by an antimalarial, artemisinin^12^,^13^. However, because the mechanisms underlying the actions of TOM are poorly understood, it is not generally used for modern healthcare, and thus, an understanding of these mechanisms would undoubtedly increase its medical usages considering its fewer toxicities and side effects^14^. Many attempts have been made to characterize TOM using chemistry-based, target-based, and system biological approaches^15^,^16^. In particular, one method based on comparisons of structural features of TOM with those of human metabolites could explain the nontoxic aspects of its functions. Similarities between compounds and metabolites, so-called metabolite-likeness, have proven to be significant to drug discovery^17^. Actually, some TOM-derived compounds with structural features in common with metabolites have been reported to function in manners similar to structurally related metabolites. For example, bufalin which is structurally similar to vitamin D_3_, binds to vitamin D receptor and protects skin cells after UV exposure^18^. However, the functions of many TOM compounds have not been studied as yet.

Members of the monoamine oxidase (MAO) enzyme family catalyze the oxidation of monoamines to produce hydrogen peroxide, aldehydes, and substituted amines. There are two MAO isoenzymes: MAOA and MAOB. The former is known to be expressed in intestine, liver, and peripheral adrenergic neurons whereas MAOB is expressed in brain, liver, and lungs^19^. MAOA and MAOB have been viewed as therapeutic targets in neurological disorders, because they inactivate multiple catecholamines, such as dopamine, adrenaline, and serotonin^19^. In particular, selegiline and rasagiline, which bind to and inhibit MAOB, show disease-modifying effects in Parkinson’s disease^20^. However, few have suggested a role for MAOB inhibitors in the context of radiotherapy for lung cancer.

Based on previous findings, we hypothesized that TOM-derived human metabolites mimetic NF-κB inhibitors could regulate IR-induced radioresistance and EMT in NSCLC cells with lower toxicity and fewer side effects. In the present study, we analyzed structural similarities between TOM-derived compounds and human metabolites, and examined the abilities of TOM compounds to induce radiosensitizing activity in NSCLC cells. Lead compounds for developing inhibitors specific for NF-κB may exert therapeutic effects for treating radioresistant tumors.

## Results

### Identification of TOM-derived metabolite mimetic NF-κB inhibitors as candidates of radiosensitizers

To identify TOM-derived radiosensitizers, we first screened TOM-derived compounds for candidate NF-κB inhibitors. Structural information of active compounds in TOM provided by the TCM Database@Taiwan was utilized^21^. Of the active compounds observed in TOM, candidate NF-κB inhibitors were selected by searching two electronic literature databases (Pubmed and Web of Science). Using the defined criteria, this search retrieved 180 compounds. Further literature-based study resulted in the selection of 83 compounds for further study (Supplementary Table S1). To identify candidate of radiosensitizers that inhibit IR-induced NF-κB activation in NSCLC cells, the 83 selected compounds were prepared and primary screening for candidates that regulated the transcriptional activity of NF-κB in IR irradiated cells was performed using a reporter gene assay. Ultimately, 20 compounds were selected for further investigation (Supplementary Table S1).

**Figure 1:**
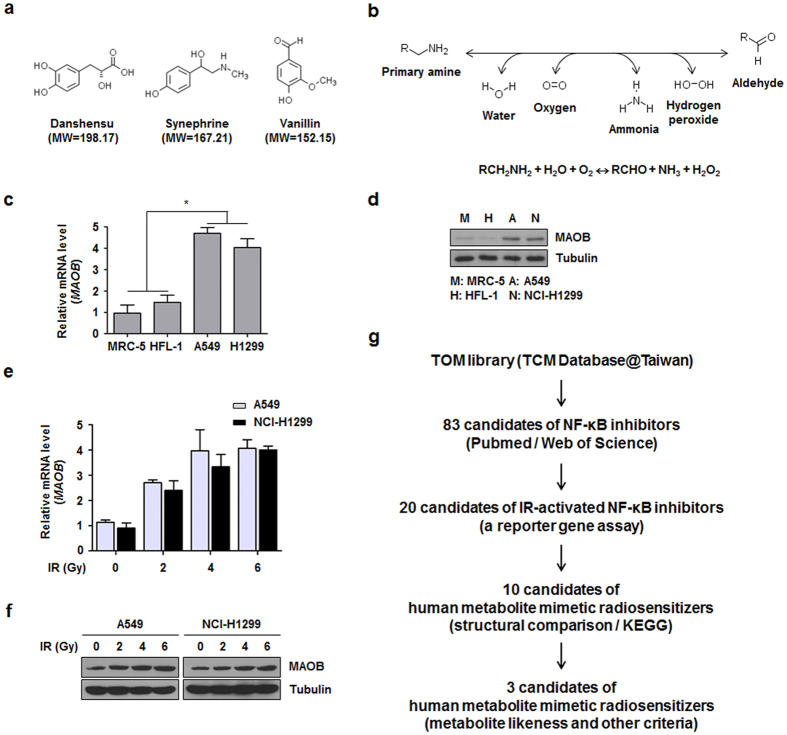
Procedures for selecting radiosensitizer candidates from TOM-derived metabolite mimetic NF-κB inhibitors. (**a**) Molecular structures of danshensu, synephrine, and vanillin, the three TOM-derived human metabolite mimetics examined during the present study. MW, molecular weight. (**b**) The chemical reaction catalyzed by MAOB. Hydrogen peroxide is produced during the reaction. (**c,d**) MAOB mRNA and protein expression levels were higher in A549 and NCI-H1299 cells (human NSCLC cell lines) than in MRC-5 and HFL-1 cells (human normal lung cell lines). *p < 0.05 when normal lung cell lines were compared with NSCLC cell lines. (**e,f**) The MAOB expression increased dose-dependently at the mRNA and protein levels in A549 and NCI-H1299 cells. (**g**) A schematic of the process used for identifying candidate of radiosensitizers among TOM-derived metabolite mimetic inhibitors of NF-κB.

A large-scale structural comparison of the 20 selected compounds against human metabolites was next performed to search for human metabolite mimetic NF-κB inhibitors. Structural information on human metabolites of the Kyoto Encyclopedia of Genes and Genomes (KEGG) were analyzed^22^. Structural comparisons were conducted using the Small Molecule Subgraph Detector^23^. The results of structural similarity analysis of the 20 selected TOM-derived compounds versus human metabolites are summarized in Supplementary Table S2. Results of extensive pairwise structural comparisons reveal that 10 compounds (2-methoxystypandrone, celastrol, synephrine, danshensu, sodium ferulate, daidzein, resveratrol, vanillin, wogonin, and indirubin) bore closest resemblances to human metabolites, that is, they had higher similarity scores (Supplementary Table S2). Such searches for metabolites structurally resembling TOM provide clues regarding targets and mechanisms of action (Supplementary Table S2), and more detailed examinations of metabolites, metabolic enzymes, and relevant pathways aid the elucidation of the roles of TOM in biological processes^15^. For instance, metabolites similar to synephrine, such as norepinephrine and dopamine appear to be involved in phenylalanine metabolism (Supplementary Fig. S1). In fact, synephrine is a final metabolic product of phenylethanolamine N-methyltransferase^24^. Interestingly, danshensu which has a structure comparable to those of metabolites involved in tyrosine metabolism, such as 4-hydroxyphenyllactate, L-DOPA, and 4-hydroxyphenylpyruvate, has been reported to inhibit tyrosine phosphorylation^25^. During such structural similarity studies, it is important to set appropriate thresholds, and thus, in the present study, we defined structural similarity as a Tanimoto similarity value of ≥0.5 (ref. 17). Based on metabolite-likeness and other criteria, such as the cellular localizations and physiological functions of target enzymes, relationships with NF-κB pathway and lung cancer, drug-likeness, and functional novelty, three TOM-derived mimetics of human metabolites were identified, namely, danshensu, synephrine, and vanillin (Fig. 1a). Human metabolites structurally similar to the three TOM compounds and their corresponding enzymes for metabolic pathways were then analyzed. We focused on MAOB which is a common metabolic enzyme of structurally similar metabolites to TOM, namely dopamine to danshensu, L-metanephrine to synephrine, and 3,4-dihydroxyphenylacetaldehyde to vanillin (Supplementary Table S2). Hydrogen peroxide which is the product of reactions MAOB catalyze (Fig. 1b), is important for NF-κB pathway in NSCLC cells^26^. We also investigated MAOB expression in normal lung cells (MRC-5 and HFL-1 cells) and NSCLC cells (A549 and NCI-H1299 cells) and found that MAOB was overexpressed in NSCLC cells as compared with normal lung cells (Fig. 1c,d). In addition, we measured the expression of MAOB in irradiated NSCLC cells. As shown in Fig. 1e,f, IR treatment dramatically and dose-dependently induced MAOB expression at the mRNA and protein levels in A549 and NCI-H1299 cells. These findings led us to hypothesize that the activation of MAOB signaling might be a new biomarker for NSCLC cells and for radioresistance involving activation of NF-κB pathway. The strategy used to identify TOM-derived metabolite mimetic NF-κB inhibitors as candidate of radiosensitizers is presented in Fig. 1g.

### Danshensu binds directly to MAOB and blocked its activity in NSCLC cells

After selecting the three TOM-derived metabolite mimetics (danshensu, synephrine, and vanillin) and MAOB as their target enzyme, we performed an isothermal titration calorimetry (ITC) experiment to compare their binding parameters with recombinant human MAOB. By analyzing ITC data, which show how much heat is generated or absorbed upon ligand-binding, binding constants and enthalpy and entropy changes can be precisely measured, and the thermodynamic properties of a molecular interaction are determined. For danshensu, calorimetric data revealed that heat was released when it associated with MAOB, indicating that these interactions made significant enthalpic contributions to binding (Supplementary Table S3). ITC findings also revealed slightly unfavorable entropic contributions, suggesting MAOB was slightly stabilized by binding. This effect was especially noticeable for the inhibitors, possibly promoted by significant reductions of *B*-values of the MAOB binding pocket when binary complexes were formed. ITC showed that MAOB bound to the mimetics with a 1:1 stoichiometry, indicating that the protein concentration could be determined with reasonable accuracy and that the protein was properly folded. As shown in Supplementary Table S3, danshensu and selegiline (a known MAOB inhibitor^27^) had dissociation constants of 16 ± 0.8 and 15 ± 2.4 μM, respectively. On the other hand, synephrine (183 ± 10.3 μM), vanillin (165 ± 9.1 μM) and other compounds with a similar flat, aromatic ring structure, such as tyrosine and phenylalanine, showed relatively weak binding to MAOB, based on ITC data.

**Figure 2:**
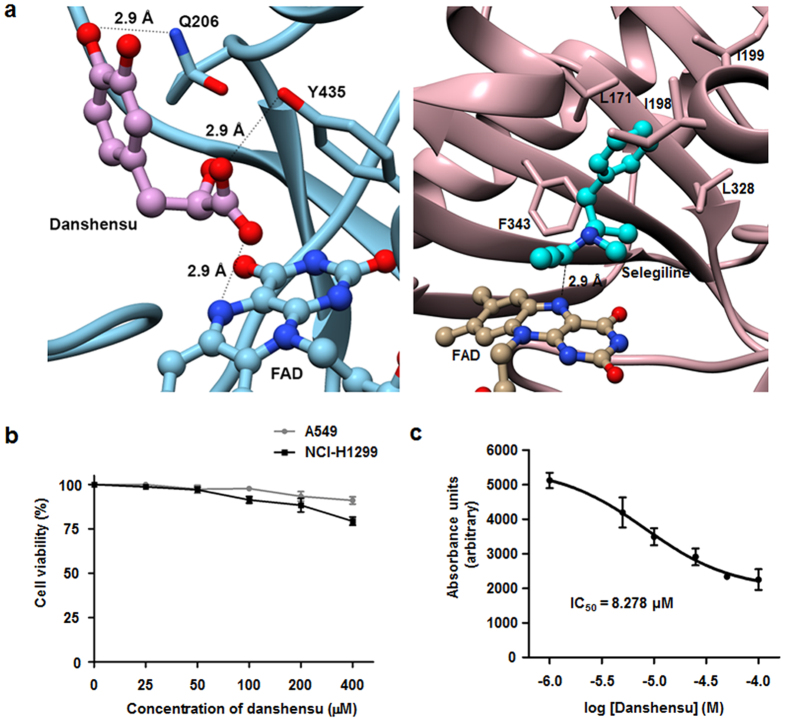
Danshensu binds directly to MAOB and diminished its activity in NSCLC cells. (**a**) Molecular docking of MAOB with danshensu or selegiline. In agreement with ITC results, danshensu and selegiline showed significant affinity for MAOB. (**b**) The effect of different concentrations of danshensu on NSCLC cell viability. Danshensu did not reduce cell viability substantially at concentrations up to 50 μM. (**c**) Inhibition of MAOB activity, danshensu had an IC_50_ of 8.278 μM.

Molecular modeling of human MAOB (2VRM) confirmed that inhibitors dock in a typical MAO binding site. An inhibitor docking experiment on selegiline, danshensu, synephrine, and vanillin returned results in-line with ITC results. Selegiline (the control) showed highest affinity for MAOB with −6.1 kcal·mol^−1^. Selegiline was positioned on top of the si-face of the isoalloxazine ring at a distance of 2.9 Å and between the hydrophobic residues Leu171, Ile198, Ile199, Leu328, and Phe343 (Fig. 2a). Of the mimetics, danshensu exhibited the highest affinity for MAOB at −5.9 kcal·mol^−1^. Danshensu formed 2.9 Å hydrogen bonds with flavin adenine dinucleotide (FAD), Gln206 and Tyr435 (Fig. 2a). The affinities of synephrine and vanillin were −4.9 and −5.6 kcal·mol^−1^, respectively. Both molecules did not show strong hydrogen bonding with MAOB (Supplementary Fig. S2).

After conducting the above studies, we examined the cytotoxicity of danshensu to determine the concentration of the compound that could be used without affecting cell viability. NSCLC cells were treated with different concentrations of danshensu or dimethyl sulfoxide (DMSO) as vehicle (Fig. 2b). It was found that danshensu did not affect cell viability at concentrations up to 50 μM, and the concentration was used in this study. Having established direct binding between MAOB and danshensu by ITC, we examined the inhibition profile for danshensu with respect to MAOB activity *in vitro*. Danshensu was found to be a fairly potent inhibitor of MAOB and inhibited its enzyme activity by 50% (IC_50_) at 8.3 μM (Fig. 2c). Under the same experimental conditions, the IC_50_ value of selegiline was 1.6 μM. Taken together, these findings indicate that danshensu significantly inhibited MAOB activity via direct binding, and imply that it might be viewed as a lead compound for the inhibition of the MAOB-mediated activation of the NF-κB pathway induced by IR irradiation.

### Danshensu blocks IR-induced NF-κB activation in NSCLC cells

**Figure 3:**
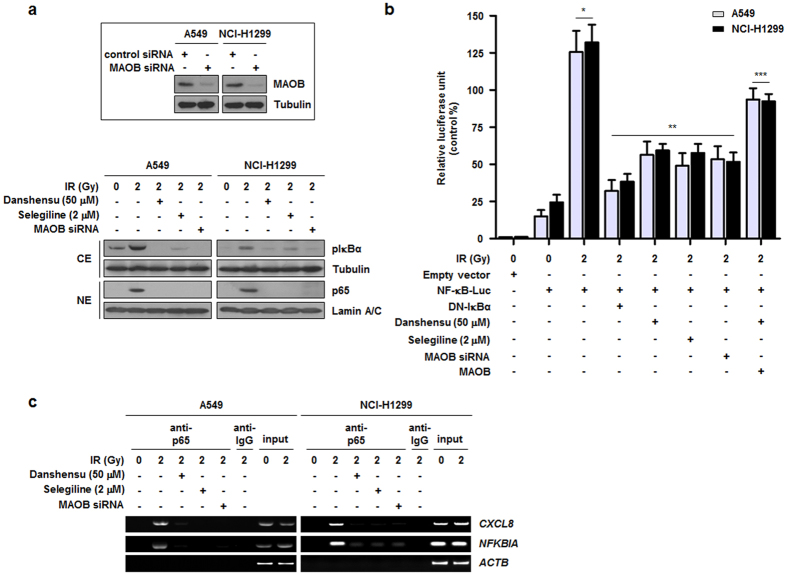
IR-induced NF-κB activation is blocked by danshensu. (**a**) Treatment with danshensu, selegiline, or MAOB siRNA inhibited IR-induced increases in IκBα phosphorylation and the nuclear translocation of p65. CE and NE refer to cytoplasmic extracts and nuclear extracts, respectively. (**b**) NF-κB-specific luciferase reporter assay indicated that the IR-induced transcriptional activation of NF-κB was reduced by treatment with danshensu, selegiline, or MAOB siRNA, and these reductions were rescued by MAOB overexpression. DN-IκBα, domininant negative IκBα which has S32/36A mutations preventing its phosphorylation. *p < 0.05 vs. non-irradiated cells; **p < 0.05 vs. irradiated cells; ***p < 0.05 vs. irradiated cells treated with danshensu. (**c**) IR-induced recruitments of p65 to the promoter regions of *CXCL8* and *NFKBIA* (the two known target genes of NF-κB) were reduced by danshensu, selegiline or MAOB-specific siRNA.

In a previous study, we suggested that IR increases the activity of NF-κB in NSCLC cells^9^,^10^,^28^. Thus, to determine whether IR-induced NF-κB activation could be regulated by danshensu, A549 and NCI-H1299 cells were treated with danshensu and 2 Gy of IR and incubated for 2 h. As shown in Fig. 3a, IR-induced IκBα phosphorylation and the nuclear translocation of p65 were detected in both NSCLC cell lines, and these increases were significantly suppressed by danshensu (50 μM). In addition, selegiline (2 μM) and siRNA specific for MAOB also significantly reduced IR-induced NF-κB activation. Furthermore, reductions of IR-induced NF-κB transcriptional activation by danshensu, selegiline, and MAOB-specific siRNA were confirmed in A549 and NCI-H1299 cells, and the inhibitory effects of danshensu on IR-induced NF-κB activation were rescued by MAOB overexpression (Fig. 3b). Next, we conducted a chromatin immunoprecipitation (ChIP) assay to verify the effect of NF-κB inhibitors on NF-κB activation, and focused on specific NF-κB binding sites within the promoters of *CXCL8* and *NFKBIA*, which are both well-known NF-κB target genes^9^,^29^. As compared to IR alone, treatment with danshensu, selegiline, or MAOB-specific siRNA significantly reduced the recruitment of p65 by *CXCL8* and *NFKBIA* promoter regions (Fig. 3c). Taken together, our data suggest that NF-κB activation induced by IR was diminished by danshensu treatment due to MAOB inhibition.

### Danshensu increases IR-induced apoptosis and radiosensitivity in NSCLC cells

**Figure 4:**
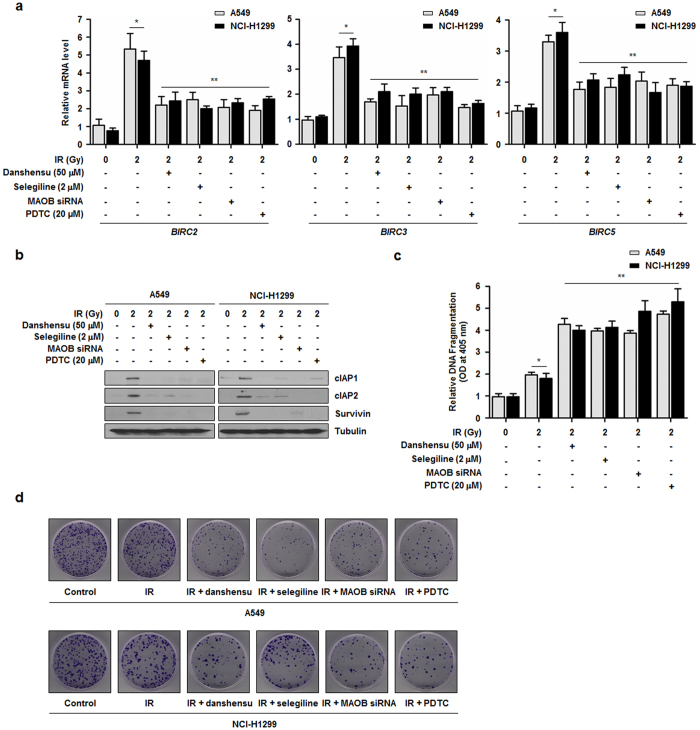
Danshensu enhances IR-induced apoptosis and the radiosensitivity of NSCLC cells. (**a,b**) The mRNA and protein expressions of three NF-κB-regulated prosurvival genes (*BIRC2*, *BIRC3*, and *BIRC5*) were significantly upregulated after IR, but these upregulations were blocked by danshensu, selegiline or MAOB siRNA. *p < 0.05 vs. non-irradiated cells; **p < 0.05 vs. irradiated cells. (**c**) Apoptotic cell death was assessed using a DNA fragmentation assay. When IR-induced MAOB and NF-κB activation were inhibited NSCLC cell viability was adversely affected. *p < 0.05 vs. non-irradiated cells; **p < 0.05 vs. irradiated cells. (**d**) The colony formation assay showed that NF-κB activation by IR or the inhibition of MAOB by 50 μM danshensu, 2 μM selegiline, and 20 μM PDTC suppressed long-term cell proliferation.

We next examined transcriptional changes of *BIRC2*, *BIRC3*, and *BIRC5* (known IR-induced NF-κB-regulated prosurvival genes^30^,^31^,^32^) induced by danshensu. Significant inductions of *BIRC2*, *BIRC3*, and *BIRC5* mRNA and concomitant overexpressions of their proteins were observed in IR treated A549 and NCI-H1299 cells, and the expressions of these genes were reduced by treatment with danshensu, selegiline or MAOB-specific siRNA (Fig. 4a,b). Next, to confirm danshensu is functionally involved in the regulation of the radiosensitization of NSCLC cells, an apoptosis assay was performed. As compared with untreated NSCLC cells, danshensu-treated cells were more sensitive to IR-mediated cytoplasmic histone-associated DNA fragmentation, a measure of apoptotic cell death (Fig. 4c). To evaluate the long-term effect of danshensu on cell proliferation, a colony formation assay was conducted. Formations of colonies by A549 and NCI-H1299 cells after IR irradiation were greatly diminished by danshensu (Fig. 4d). As shown in Fig. 4c,d, the involvement of MAOB in the NF-κB-mediated cell survival pathways was confirmed by treating NSCLC cells with selegiline or MAOB-specific siRNA. In addition, cells were treated with 20 μM pyrrolidine dithiocarbamate (PDTC, a NF-κB inhibitor) to confirm that NF-κB directly affects anti-apoptotic pathways activated by IR (Fig. 4a–d). The results obtained suggested that danshensu reduced prosurvival gene expression, regulated IR-induced apoptosis, and ultimately conferred radiosensitization by inhibiting MAOB activity in both NSCLC cell lines.

### Danshensu reduces cancer-associated inflammation in response to IR in NSCLC cells

**Figure 5:**
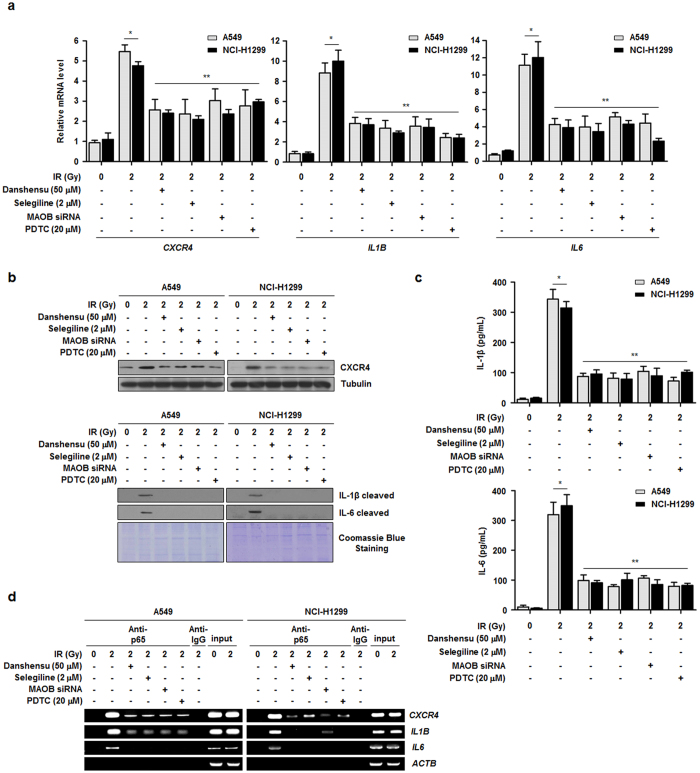
Danshensu reduces IR-induced inflammation mediated by NF-κB. (**a**) The transcriptional upregulation of NF-κB-regulated proinflammatory genes by IR was inhibited by treatement with danshensu, selegiline, MAOB siRNA, or PDTC. *p < 0.05 vs. non-irradiated cells; **p < 0.05 vs. irradiated cells. (**b**) Cellular CXCR4, IL-1β, and IL-6 expressions after IR treatment were assessed by Western blotting. Danshensu, selegiline, MAOB siRNA, and PDTC reduced their expressions after IR treatment. Coomassie blue staining was used to confirm equal protein loading. (**c**) The effects of MAOB and NF-κB inhibition on the secretions of IL-1β and IL-6 by irradiated NSCLC cells were evaluated by ELISA. *p < 0.05 vs. non-irradiated cells; **p < 0.05 vs. irradiated cells. (**d**) p65 recruitments to the promoter regions of *CXCR4*, *IL1B*, and *IL6* were confirmed using a ChIP assay.

We next examined IR-induced transcriptional changes of *CXCR4*, *IL1B*, and *IL6*, which are all proinflammatory genes regulated by NF-κB^10^,^32^,^33^. Significant reductions in the mRNA levels of *CXCR4*, *IL1B*, and *IL6* were observed in NSCLC cells that had been treated with danshensu and IR (Fig. 5a). Concomitantly, the productions of cellular CX-C chemokine receptor type 4 (CXCR4) and the secreted active forms of interleukin (IL)-1β and IL-6 were reduced by danshensu (Fig. 5b). Under the same experimental conditions, reduced secretion of both IL-1β and IL-6 was measured by a cytokine-specific enzyme-linked immunosorbent assay (ELISA) (Fig. 5c). To verify the direct association of NF-κB inhibitors and the expression profiles of proinflammatory genes, we conducted an additional ChIP assay. As compared to IR alone, co-treatment with IR and danshensu caused a significant decrease in the recruitment of p65 to the promoter regions of *CXCR4*, *IL1B*, and *IL6* (Fig. 5d). In addition, the involvement of MAOB in NF-κB-mediated proinflammatory pathways was confirmed by treating cells with selegiline, MAOB-specific siRNA, or PDTC (Fig. 5a–d). These results show that danshensu diminished the expressions of proinflammatory genes by reducing NF-κB binding to promoters.

### Danshensu prevents IR-induced EMT in NSCLC cells

**Figure 6:**
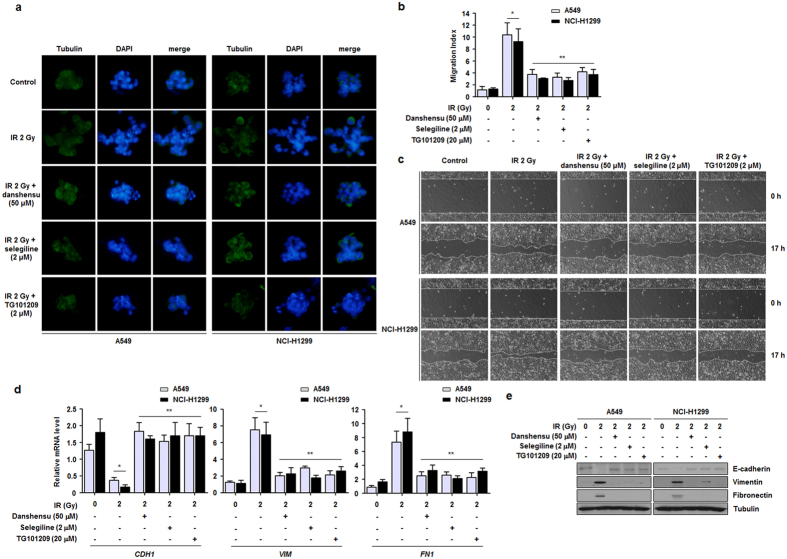
IR-induced EMT is prevented by danshensu in NSCLC cells. (**a**) Morphological modifications observed in a 3D culture system suggested that IR-induced EMT was inhibited by danshensu and selegiline. (**b,c**) MAOB inhibition by danshensu significantly decreased irradiated NSCLC cell mobility as determined by the transwell cell migration and wound healing assays. *p < 0.05 vs. non-irradiated cells; **p < 0.05 vs. irradiated cells. (**d,e**) The mRNA and protein levels of EMT markers (E-cadherin-an epithelial marker, Vimentin and Fibronectin-mesenchymal markers) were assessed by qRT-PCR and Western blotting. Danshensu was found to reduce IR-induced EMT. *p < 0.05 vs. non-irradiated cells; **p < 0.05 vs. irradiated cells.

Several lines of evidence have shown that NF-κB and inflammation influence EMT and metastasis^10^,^28^. To explore the potential effects of danshensu on inflammation-mediated metastatic conversion, we monitored morphological changes in both NSCLC cell lines. The 3D culture model has been used to identify the morphological modifications which are characteristic of cancer growth^34^. In this system, IR-treated NSCLC cells could be distinguished from control cells and formed more acini that invaded the Matrigel 3D matrix. However, these morphological changes were abrogated by danshensu, indicating that inhibition of NF-κB activity by danshensu in NSCLC cells prevented IR-dependent EMT (Fig. 6a). We then measured the migration abilities and EMT marker protein expressions in NSCLC cells treated with danshensu, and danshensu was found to reduce motility, as determined by transwell cell migration and the wound healing assay (Fig. 6b,c). A Janus kinase 2 inhibitor (TG101209) was also administered as a positive EMT inhibitor control^34^. Danshensu treatment was found to prevent IR-induced EMT by increasing the expression of E-cadherin (an epithelial marker) and decreasing the expressions of Vimentin and Fibronectin (mesenchymal markers) at the mRNA and protein levels (Fig. 6d,e). Consequently, our results suggest that danshensu suppresses IR-induced metastatic conversion in NSCLC cells.

### Danshensu increases *in vivo* radiosensitization and decreases *in vivo* EMT in a xenograft mouse model

**Figure 7:**
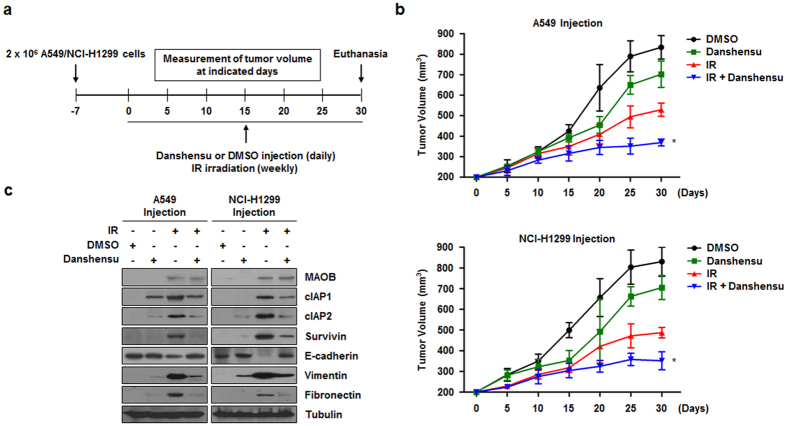
Danshensu induces *in vivo* radiosensitization and decreases EMT in a mouse xenograft model. (**a**) A schematic of the procedure used to investigate the *in vivo* radiosensitizing effects of danshensu. (**b**) Graphs showing effects of danshensu on the volumes of tumor xenografts. *p < 0.05 vs. mean tumor volume at day 30 in mice administered irradiation alone. (**c**) Tumor tissue lysates of danshensu-pretreated mice indicated the expressions of pro-survival and EMT-related proteins induced by IR were reduced by danshensu.

Since danshensu was found to have a radiosensitizing and EMT-inhibitory effect in NSCLC cells, we further examined these effects *in vivo*. To evaluate the combined effects of danshensu and IR on tumor growth *in vivo*, a xenograft mouse model was established (Fig. 7a). *In vivo* data from nude mice bearing tumors formed by A549 or NCI-H1299 cells indicated that danshensu conferred *in vivo* radiosensitization (Fig. 7b). The tumor volumes of mice treated with IR and danshensu were significantly reduced by 30.3% (for A549 cells) or by 27.9% (for NCI-H1299 cells) at 30 days after IR and danshensu treatment as compared with mice treated with IR alone. In addition, the IR-induced expressions of prosurvival proteins and of EMT-related proteins were considerably lower in the extracted tumor tissue lysates when danshensu was administered directly at tumor sites (Fig. 7c). Taken together, these findings suggest that danshensu significantly increases *in vivo* radiosensitization and inhibits EMT.

## Discussion

TOM is used to treat multiple cancer types, prevent cancer, and regulate tumor development with fewer side effects^14^. TOM has been reported to improve efficacy of chemotherapy and prolong patient survival in NSCLC^35^. However, few previous studies or clinical trials have described target-based approaches that aid the identification of TOM-derived compounds enhancing therapeutic impact. Here, by searching web databases and conducting a reporter gene assay, we were able to select three candidate potential radiosensitizing agents for NSCLC from numerous TOM compounds. Structural comparisons of human metabolites and TOM provide a systems-based means of identifying effective therapeutic strategies^15^. These comparisons led to the identification of human metabolites with structures similar to the three candidates, and to the identification of target enzymes (Supplementary Table S2). Of the several target enzymes identified, monoamine oxidase, especially its type B isoenzyme, caught our interest due to its expression in lung tissues^36^. Our ITC and molecular modeling results suggested that danshensu was the only compound among the three final candidates that would exhibit significant affinity for MAOB (Fig. 2a and Supplementary Table S3), and thus, danshensu was selected for additional experiments and found to show considerable inhibitory effects on IR-activated MAOB and NF-κB activities in NSCLC cells.

Because MAOs inactivate catecholamine neurotransmitters, including adrenaline and dopamine, the inhibitions of their activities have been viewed as a means of achieving neuroprotective effects. When isoform-selective inhibitors were developed, the inhibitors of MAOA and MAOB were found to exhibit antidepressant effects and therapeutic effects in Parkinson’s diseases, respectively, and thus, subsequent studies on MAOB were centered on their roles in neurons, glial cells, and in neurodegenerative diseases^19^. Although MAOB is known to be expressed in pulmonary tissues, its function in lungs and the clinical implication of its expression in lung cancer have not been determined. In the present study, we suggest for the first time that inhibiting MAOB activity could alleviate radioresistance by inactivating NF-κB in NSCLC cells. The present study shows that MAOB inhibition reduces NF-κB activity in lung cancer cells, whereas in neuronal cells treatment of MAOB inhibitors such as phenelzine and selegiline has been reported to upregulate or not affect NF-κB signals, respectively^37^,^38^. Furthermore, we observed that the previously known MAOB inhibitor selegiline reduced activation of NF-κB and EMT in irradiated NSCLC cells, and according to a previous study, it protects nontumorigenic cells from IR^39^. Intriguingly, danshensu was also found to increase survival of MRC-5 and HFL-1 cells (normal lung cells) in response to IR irradiation, according to our data (Supplementary Fig. S3). These results imply that inhibiting MAOB may be a promising strategy for enhancing efficacy of radiotherapy.

Danshensu is a hydrophilic component of *Salvia miltiorrhiza* Bunge, and is also known to inhibit platelet aggregation and to have antioxidant properties^40^. The present study shows that danshensu directly binds MAOB and suppresses its activity in IR-treated NSCLC cells. When we examined how danshensu physically associates with MAOB, we found that it binds to its substrate cavity. A substrate cavity (420 å^3^) and an entrance cavity (290 å^3^) constitute the active site and these two cavities are interconnected by an Ile199 side chain. Danshensu was found to form a hydrogen bond with N5 atom of FAD, like all other irreversible MAOB inhibitors found to date^41^. Tyr398 and Tyr435 of MAOB form an aromatic cage that aids recognition of the substrate amino group^42^. In particular, Tyr435 polarizes the substrate amine and guides it toward the flavin ring of MAOB, which significantly contributes to its enzyme activity^43^. According to our molecular modeling data, danshensu forms a hydrogen bond with Tyr435 of MAOB, but synephrine and vanillin do not (Fig. 2a). This could be a ground for only danshensu binding and having a functional relationship with MAOB. Furthermore, the antioxidant activity of danshensu may inhibit H_2_O_2_ which can be the by-product of reactions catalyzed by MAOB. It was reported that H_2_O_2_ could induce NF-κB activation^44^. Considering antioxidant properties of danshensu, it may inhibit IR-induced NF-κB signal by decreasing H_2_O_2_ formation. Nevertheless, in the present study, NF-κB-inhibitory effects of danshensu on irradiated NSCLC cells were similar to those of selegiline and MAOB siRNA. This indicates that MAOB inhibition of danshensu by direct binding, rather than H_2_O_2_ inhibition by danshensu, was a major cause of the reduction in NF-κB activity in IR-irradiated NSCLC cells.

The present study confirms that the mRNA and protein expressions of MAOB are higher in NSCLC cells than in normal lung cells (Fig. 1c,d) and are further increased dose-dependently by IR treatment (Fig. 1e,f), which has not been previously reported. Thus, because the inhibition of MAOB by danshensu, selegiline, or MAOB siRNA blocked radioresistance in NSCLC cells, we suggested the possibility that MAOB may be considered as a biomarker of NSCLC radiation responses. Monoamines like serotonin and L-DOPA are known to regulate the bystander effects of IR irradiation, and these monoamines may be located at membrane receptors in response to IR^45^. We suggest further longitudinal investigation be conducted to examine MAO-associated post-IR exposure events and their clinical implications.

According to our luciferase reporter gene assay data (Fig. 3b), danshensu significantly reduced NF-κB transcriptional activity. Interestingly, the overexpression of MAOB increased the transcriptional activity of NF-κB in IR- and danshensu-cotreated cells, but its level did not reach that of cells treated with radiation alone. This indicates that target molecules other than MAOB and NF-κB are likely to be involved in the downstream pathway initiated by danshensu. Most TOM compounds have multiple targets and as the human body possesses innumerable factors that influence drug response, drug development groups in the West are increasingly adopting polypharmacologic approaches^46^. As regards the development of TOM-derived drugs, the present study provides yet another example that drug potency is often affected by several signaling molecules.

In the present study, we searched for TOM candidates that inhibit IR-induced NF-κB activation in NSCLC cells by utilizing online databases and comparing structural properties to those of human metabolites. Danshensu and MAOB were chosen as the TOM candidate and target enzyme, respectively. Danshensu was found to significantly inhibit IR-induced MAOB activity. Furthermore, danshensu and the known MAOB inhibitor, selegiline, both downregulated NF-κB signaling and the development of aggressive phenotypes in IR-treated NSCLC cells. In addition, danshensu induced *in vivo* radiosensitization and decreased EMT of mouse xenograft models. Taken together, these findings suggest that danshensu elicits the radiosensitization of NSCLC by inhibiting its target enzyme, MAOB, and thus, reducing NF-κB activation. The results of this study provide novel insights regarding the selection of TOM candidates useful for treating NSCLC in patients undergoing radiotherapy.

## Methods

### Cell lines, cell culture, and irradiation

A549 and NCI-H1299 cell lines were obtained from the American Type Culture Collection (Manassas, VA), authenticated, and maintained no longer than 6 months after receipt. Cells were grown in RPMI-1640 containing 10% FBS, 100 U/mL penicillin, and 100 μg/mL streptomycin at 37 °C in a humidified 95% air/5% CO_2_ atmosphere. Cells were exposed to a single dose of γ-rays employing a Gamma Cell-40 Exactor (Nordion International, Inc., Kanata, Ontario, Canada) at a rate of 0.81 Gy/min. Control cells were placed in the irradiation chamber but not irradiated.

### Real-time quantitative RT-PCR (qRT-PCR)

Gene expression levels were assessed by real time qRT-PCR as described previously^47^,^48^ with several modifications. Detailed procedure is demonstrated in Supplementary Materials and Methods.

### Western blot analysis and transient transfection

For Western blot analysis, whole cell lysates were prepared as previously described^49^,^50^. Detailed procedure is demonstrated in Supplementary Materials and Methods.

### Literature search

Literature searches of the PubMed (http://www.ncbi.nlm.nih.gov/pubmed) and Web of Science (http://wokinfo.com/) databases were conducted to identify articles that assessed the inhibitory effect of TOM on NF-κB using the following keywords: (1) TOM or TCM (Traditional Chinese Medicine), (2) NF-κB, and (3) cancer or tumor. All eligible articles that examined this inhibitory effect of TOM were extracted. Initially, abstracts and titles were analyzed to identify studies on the association between TOM and NF-κB inhibition. Abstracts of studies that met this criterion were carefully read, and the full texts were analyzed using the following criteria: (1) inhibition of NF-κB transcriptional activity evaluated by a reporter gene assay; (2) comparison of the expression levels of NF-κB components in TOM-treated cells versus non-treated control; (3) written in English; (4) the applications of TOM at non-cytotoxic levels; (5) the provision of sufficient supporting data regarding NF-κB inhibition by TOM; (6) when several reports were published on the same TOM in more than one journal, the most complete study was selected for analysis; (7) studies that provided only *in vitro* analysis data were excluded. Using these criteria, 83 compounds were included for next study.

### Luciferase reporter gene assay

NF-κB-specific luciferase reporter assays were conducted to measure the transcriptional activity of NF-κB. Cells (6 × 10^5^) were plated in 6-well plates and grown to 80% confluence. To evaluate NF-κB pathway activation, cells were transiently transfected with 3 μg of NF-κB luciferase reporter gene (NF-κB-Luc) plasmid and dominant negative IκBα (DN-IκBα) plasmid using the Lipofectamine 2000 (Invitrogen, Carlsbad, CA). Following overnight transfection, luciferase reporter gene assays were carried out as described previously^9^.

### Statistical analysis

All numeric data are presented as the means ± SEMs of at least three independent experiments. Results were analyzed using one-way ANOVA for ranked data followed by Tukey’s range test, and using two-way ANOVA for ranked data followed by Bonferroni’s post hoc tests. The statistical analysis was conducted using Prism 5 software (GraphPad Software), and *p*-value of <0.05 was considered statistically significant.

## Additional Information

**How to cite this article**: Son, B. *et al.* Inhibitory effect of traditional oriental medicine-derived monoamine oxidase B inhibitor on radioresistance of non-small cell lung cancer. *Sci. Rep.* **6**, 21986; doi: 10.1038/srep21986 (2016).

## Supplementary Material

Supplementary Information
