# Supplementary material for: Inhibitory effect of traditional oriental medicine-derived monoamine oxidase B inhibitor on radioresistance of non-small cell lung cancer
Source: Sci Rep. 2016 Feb 24;6:21986. doi: 10.1038/srep21986 (PMC4764943; doi:10.1038/srep21986)
Supplement: Supplementary Information [file srep21986-s1.pdf]

Supplementary Information

Inhibitory effect of traditional oriental medicine-derived monoamine oxidase B inhibitor on radioresistance of non-small cell lung cancer

Beomseok Son[1,*], Se Young Jun[2,*], HyunJeong Seo[1], HyeSook Youn[3,†], Hee Jung Yang[4], Wanyeon Kim[3,4], Hyung Kook Kim[5], ChulHee Kang[2], and BuHyun Youn[1,2,3,4]

[1]Department of Integrated Biological Science, Pusan National University, Busan, Republic of Korea. [2]Department of Chemistry, Washington State University, Pullman, Washington, USA. [3]Nuclear Science Research Institute, Pusan National University, Busan, Republic of Korea. [4]Department of Biological Sciences, Pusan National University, Busan, Republic of Korea. [5]Department of Nanomaterial Engineering and Nanoconvergence Technology, Pusan National University, Miryang, Republic of Korea.

[†]Present address: Department of Integrative Bioscience and Biotechnology, Sejong University, Seoul, Republic of Korea.

[*]These authors contributed equally to this work.

Correspondence and requests for materials should be addressed to B.Y. (email: bhyoun72@pusan.ac.kr)

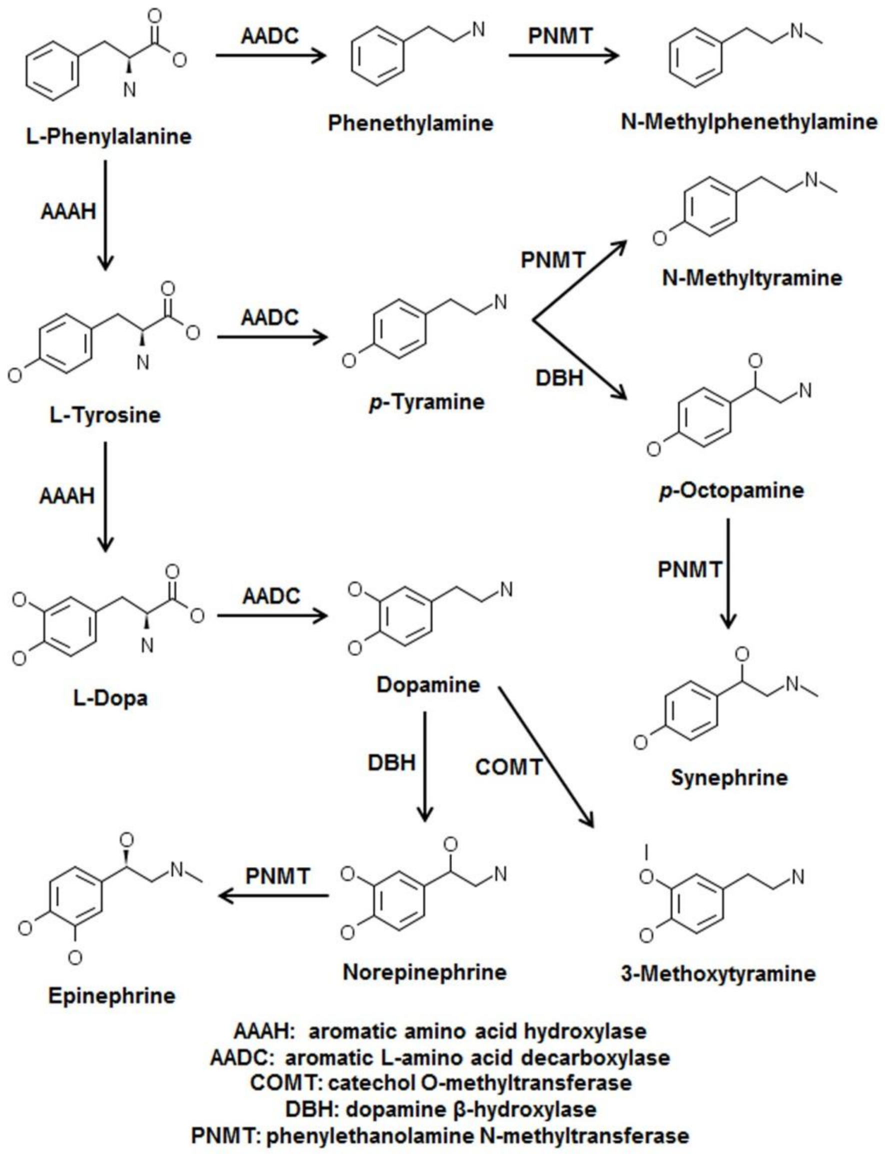

Supplementary Figure S1. Norepinephrine and dopamine which have similar structures to synephrine is involved in phenylalanine metabolism.

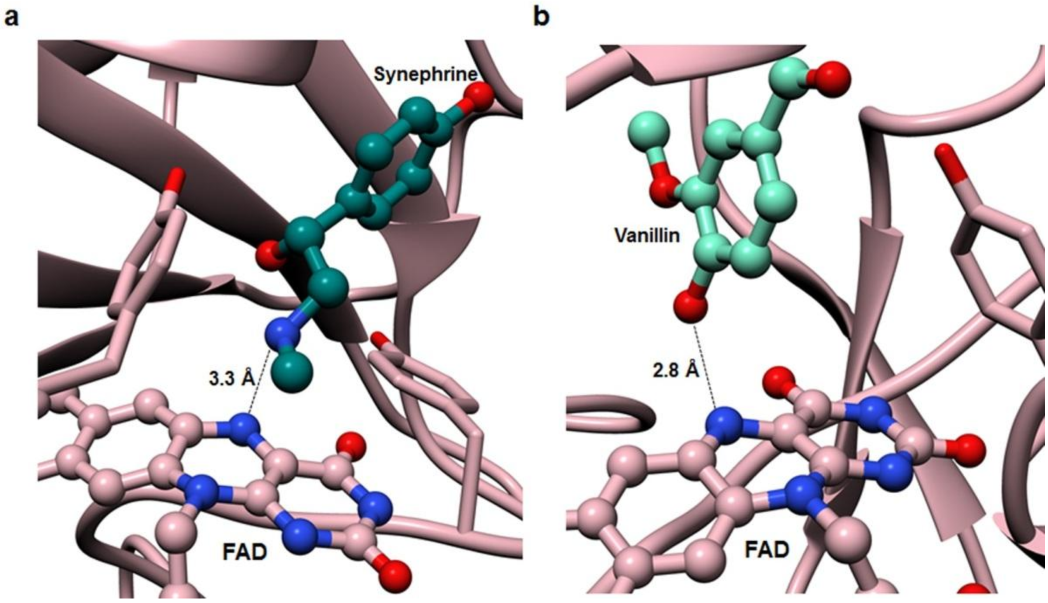

Supplementary Figure S2. Synephrine and vanillin showed relatively weak binding to MAOB.

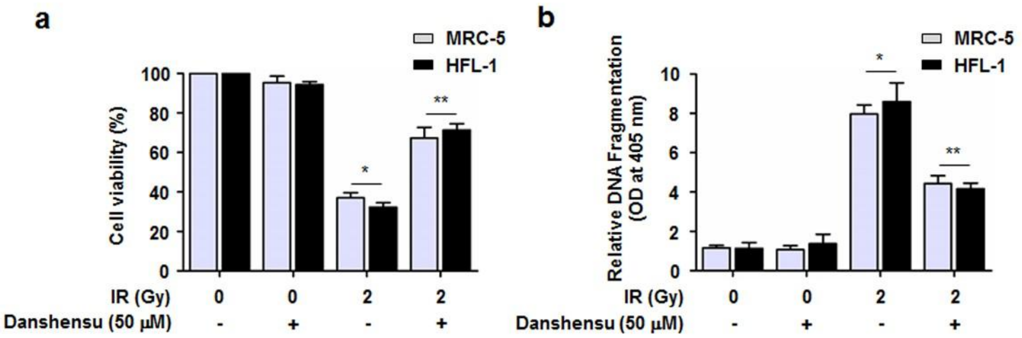

Supplementary Figure S3. Danshensu alleviated cell death induced by IR. The effect of danshensu on survival of MRC-5 and HFL-1 cells (normal lung cells) was confirmed by thiazolyl blue tetrazolium bromide assay (a) and DNA fragmentation assay (b).

Supplementary Table S1. A list of TOM-derived compounds which have inhibitory activity of IR-induced NF-κB activation in NSCLC cells.

#	Name of TOM-derived compound	Structure	NF-κB inhibitory activity (a representative reference)	IR-induced NF-κB inhibition activity (reporter assay)
1	bufalin	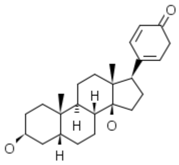	Inflammation 37(4):1050-8.	
2	N-trans-caffeoyltyramine	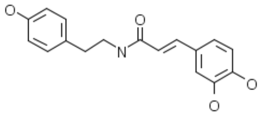	J Ethnopharmacol 28;152(3):470-7.	
3	α-L-rhamnopyranosyl-(1→6)-β-D-glucopyranosyl-3-indolecarbonate (RG3I)	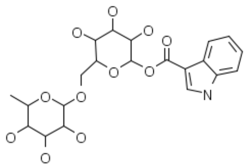	Neurochem Int 67:39-45.	
4	**2-methoxysty pandrone**	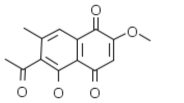	Cancer Sci 105(4):473-80.	●
5	acteoside (verbascoside)	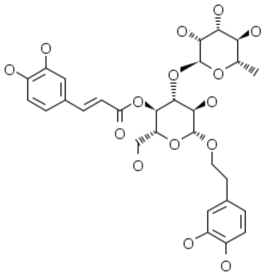	PLoS One 8(12):e80873.	
6	icariin	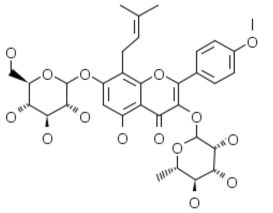	Indian J Exp Biol 51(4):313-21.	
7	farrerol	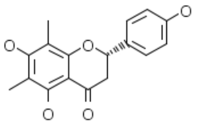	Microb Pathog 65:1-6.	

8	baicalein	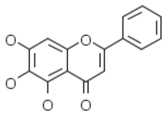	Inflammation 36(6):1584-91.	
9	piperine	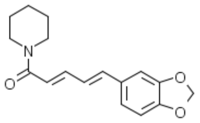	Int Immunopharmacol 17(2):293-9.	
10	**isorhamnetin**	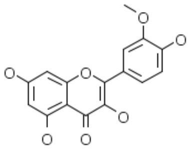	Food Chem Toxicol 59:362-72.	●
11	bisabolangelone	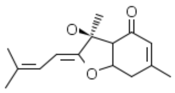	Food Chem Toxicol 59:26-33.	
12	saponin	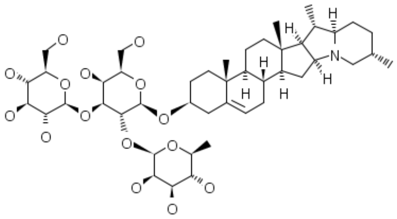	Int J Mol Med 31(6):1357-66.	
13	α-cyperone	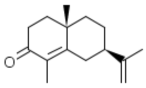	J Ethnopharmacol 147(1):208-14.	
14	echinocystic acid	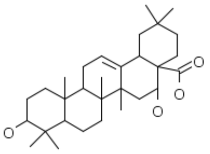	Int Immunopharmacol 15(2):433-41.	
15	eriocalyxin B	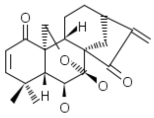	Proc Natl Acad Sci U S A 110(6):2258-63.	
16	**eupatilin**	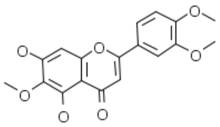	Tumour Biol 34(2):875-85.	●
17	diosgenin	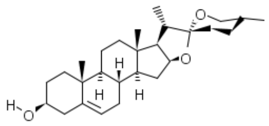	Int Immunopharmacol 15(2):240-5.	
18	effusanin C	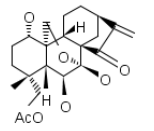	Int Immunopharmacol 15(1):84-8.	

19	capillarisin	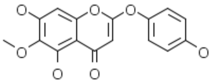	Immunopharmacol Immunotoxicol 35(1):34-42.	
20	Nitidine chloride	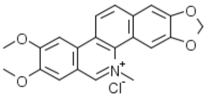	J Ethnopharmacol 144(1):145-50.	
21	betaine	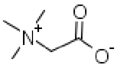	Int J Oncol 41(5):1879-85.	
22	saikosaponin a	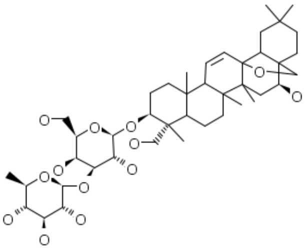	Int Immunopharmacol 14(1):121-6.	
23	pinocembrin	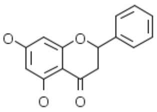	Int Immunopharmacol 14(1):66-74.	
24	puerarin	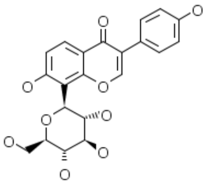	Free Radic Biol Med 53(2):357-65.	
25	**osthole**	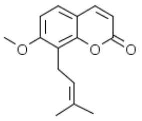	Toxicol Appl Pharmacol 261(1):105-15	●
26	matrine	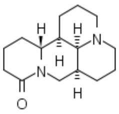	Eur J Pharm Sci 44(5):573-9.	
27	hesperidin	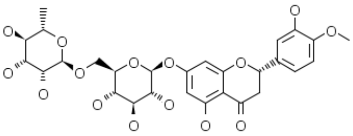	Phytother Res 26(5):657-62.	
28	**celastrol**	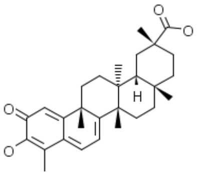	Cell Physiol Biochem 28(2):175-84.	●

29	sinomenine	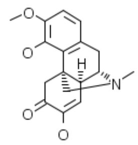	J Ethnopharmacol 137(1):457-68.	
30	rosmarinic acid	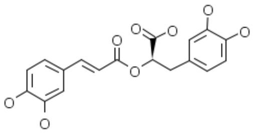	PLoS One 6(4):e18815.	
31	crocin	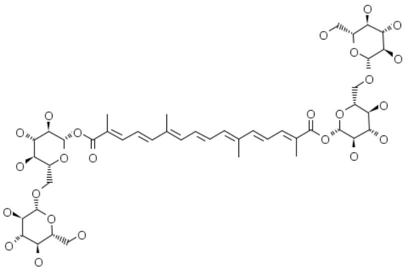	Eur J Pharmacol 648(1-3):110-6.	
32	crocetin	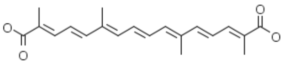	Eur J Pharmacol 648(1-3):110-6.	
33	tetrahydropalmatine	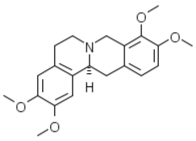	J Med Food 13(5):1125-32.	
34	methylglyoxal	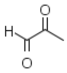	Chem Biol Interact 188(1):86-93.	
35	armepavine	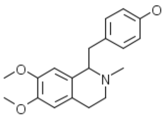	Rheumatology (Oxford) 49(10):1840-51	
36	limonene	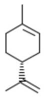	J Food Sci 75(3):H87-92.	
37	genipin	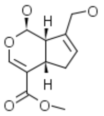	Int Immunopharmacol 10(4):493-9.	
38	baicalin	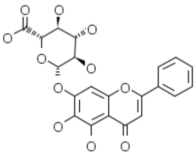	Inflammation 33(3):157-65.	

39	bigelovin	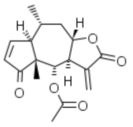	J Ethnopharmacol 123(2):250-6.	
40	pimaradienoic acid	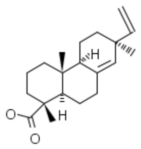	Eur J Pharmacol 601(1-3):179-85.	
41	menthone	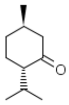	Chin J Physiol 51(3):160-6.	
42	shikonin	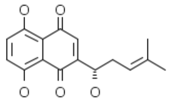	Neuropharmacology 55(5):819-25.	
43	phylligenin	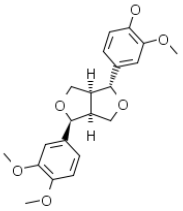	J Ethnopharmacol 118(1):113-7.	
44	kaempferol	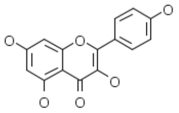	Biogerontology 8(4):399-408.	
45	1α,25-Dihydroxyvitamin D3	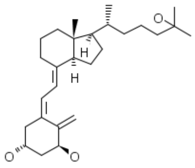	Mol Immunol 47(9):1728-38.	
46	**synephrine**	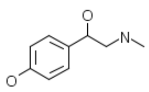	Inflamm Res 63(6):429-39.	●
47	paeoniflorin	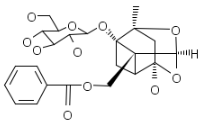	Biomed Pharmacother 62(9):659-66.	
48	**rhein**	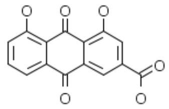	Oral Oncol 45(6):531-7.	●
49	**emodin**	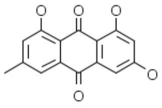	PLoS One 8(3):e57015.	●

50	**danshensu**	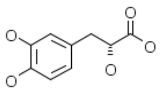	Eur J Pharmacol 708(1-3):8-13.	●
51	20(S)-Protopanaxatriol	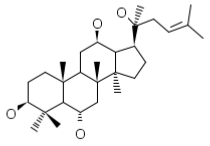	Cancer Lett 205(1):23-9.	
52	ginsenoside Rb1	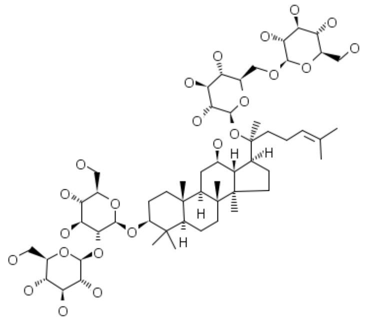	PLoS One 9(2):e87810.	
53	berberine	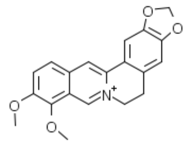	PLoS One 8(7):e69240.	
54	curculigoside A	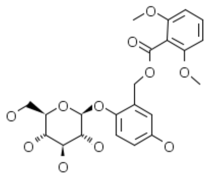	Neuroscience 192:572-9.	
55	paeonol	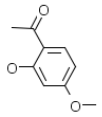	Eur J Pharmacol 588(1):124-33.	
56	**sodium ferulate**	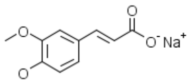	Tumour Biol 34(1):251-9.	●
57	oxymatrine	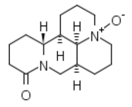	Oncol Rep 30(2):589-95.	
58	genistein	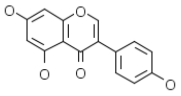	Cancer 115(10):2165-76.	
59	**daidzein**	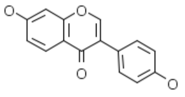	Int J Cancer 124(7):1675-84.	●

60	**epigallocatechin-3-gallate**	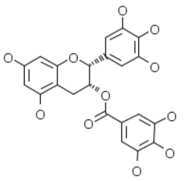	Curr Mol Med 12(2):163-76.	●
61	ascorbic acid	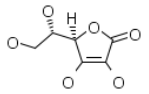	Free Radic Biol Med 68:302-14.	
62	**bilobalide**	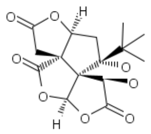	Acta Pharmacol Sin 29(5):539-47.	●
63	**hypericin**	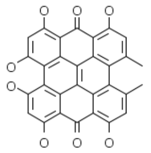	J Biol Chem 278(52):52231-9.	●
64	**resveratrol**	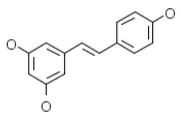	J Radiat Res 46(4):387-93.	●
65	γ-Terpinene	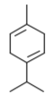	Food Chem Toxicol 57:126-31.	
66	rutin	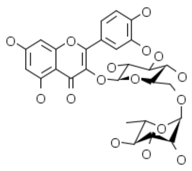	Free Radic Biol Med 69:249-57.	
67	gambogic acid	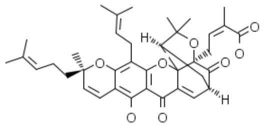	Br J Cancer 110(2):341-52.	
68	lycopene	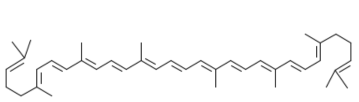	Carcinogenesis 31(10):1813-21.	
69	**vanillin**	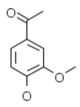	Mol Pharmacol 75(1):151-7.	●
70	gingerol	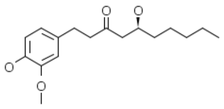	Oncogene 24(15):2558-67.	

71	diallyl sulfide	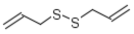	Mol Cell Biochem 311(1-2):157-65.	
72	quercetin	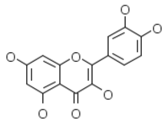	Biol Pharm Bull 36(6):944-51.	
73	myricetin	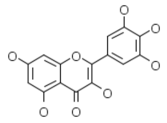	Cancer Res 68(14):6021-9.	
74	γ-tocopherol	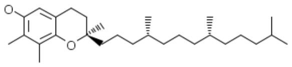	J Biol Chem 282(1):809-20.	
75	**wogonin**	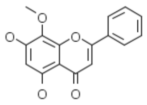	Cancer Sci 102(4):870-6.	●
76	**kavain**	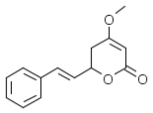	Biochem Pharmacol 71(8):1206-18.	●
77	**dihydrokavain**	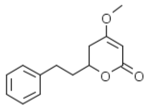	Biochem Pharmacol 71(8):1206-18.	●
78	**indirubin**	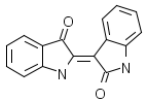	J Biol Chem 281(33):23425-35.	●
79	tanshinone IIA	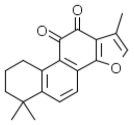	J Cell Biochem 114(9):2061-70.	
80	caffeine	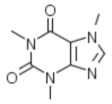	Anticancer Res 32(9):3643-9.	
81	curcumin	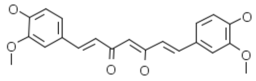	Clin Cancer Res 11(20):7490-8.	
82	triptolide	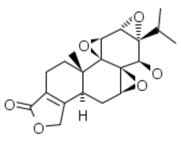	Exp Mol Med 34(6):462-8.	
83	evodiamine	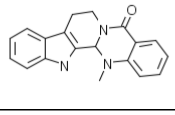	J Biol Chem 280(17):17203-12.	

Supplementary Table S2. A list of putative TOM-derived NF-κB inhibitors which are structurally similar to human metabolites (Tanimoto scores greater than or equal to 0.5 and less than 1.0).

#	Name of TOM-derived compound	Structure	Structurally similar metabolites	Tanimoto score	Putative metabolic target
1	**2-methoxystypandrone**	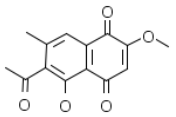	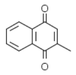 vitamin K3	0.6	vitamin K epoxide reductase
			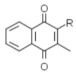 vitamin K	0.57	
2	isorhamnetin	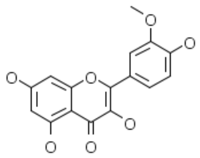	N/A		
3	eupatilin	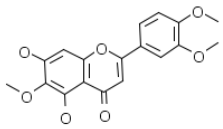	N/A		
4	osthole	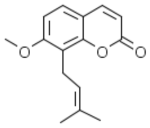	N/A		
5	**celastrol**	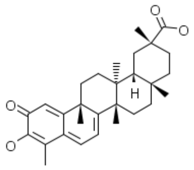	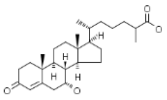 7alpha-Hydroxy-3-oxo-4-cholestenoate	0.78	cholest-5-ene-3β, 7α-diol 3β-dehydrogenase, 3alpha-hydroxyglycyrrhetinate dehydrogenase
			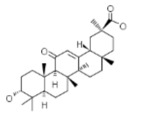 3alpha-Hydroxyglycyrrhetinate	0.72	
6	**synephrine**	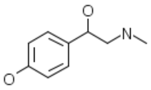	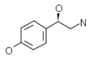 Octopamine	0.92	octopamine hydrolyase, monoamine oxidase, adrenaline oxidase
			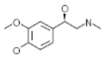 L-Metanephrine	0.86	

			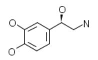 Norepinephrine	0.85	
			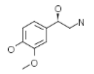 L-Normetanephrine	0.79	
			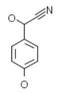 4-Hydroxymandelonitrile	0.77	
			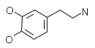 Dopamine	0.77	
			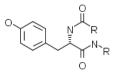 Glycogenin	0.61	
7	rhein	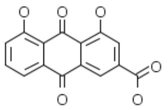	N/A		
8	emodin	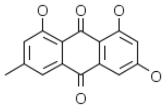	N/A		
9	**danshensu**	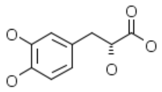	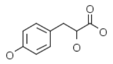 4-Hydroxyphenyllactate	0.93	hydroxyphenylpyruvate reductase, monophenol oxidase, monoamine oxidase
			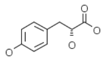 (R)-3-(4-Hydroxyphenyl)lactate	0.93	
			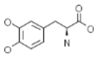 L-DOPA	0.87	
			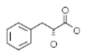 Phenyllactate	0.86	
			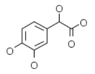 3,4-Dihydroxymandelate	0.80	

			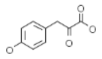<br>4-Hydroxyphenylpyruvate	0.80	
			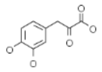<br>3,4-Dihydroxyphenylpyruvate	0.75	
			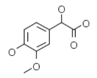<br>Vanillylmandelic acid	0.75	
			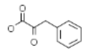<br>Phenylpyruvic acid	0.73	
			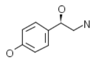<br>Octopamine	0.67	
			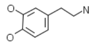<br>Dopamine	0.56	
10	**sodium ferulate**	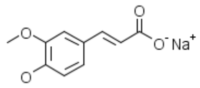	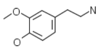<br>3-Methoxytyramine	0.50	catechol methyltransferase
11	**daidzein**	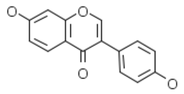	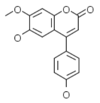<br>Melannin	0.67	tyrosinase
12	epigallocatechin-3-gallate	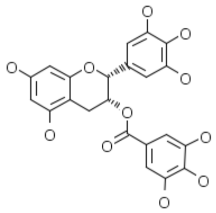	N/A		
13	bilobalide	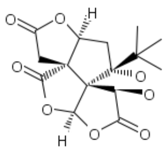	N/A		
14	hypericin	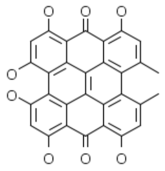	N/A		

15	**resveratrol**	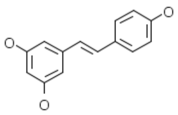	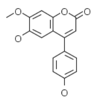 Melannin	0.58	tyrosinase
16	**vanillin**	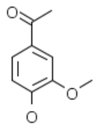	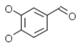 Protocatechualdehyde	0.83	aldehyde dehydrogenase, aromatic amine dehydrogenase, monoamine oxidase
			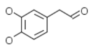 3,4-Dihydroxyphenylacetaldehyde	0.77	
			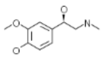 L-Metanephrine	0.73	
			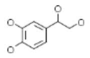 3,4-Dihydroxyphenylethyleneglycol	0.71	
			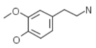 3-Methoxytyramine	0.60	
			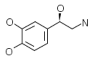 L-Noradrenaline	0.60	
			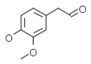 Homovanillin	0.60	
			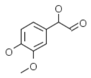 3-Methoxy-4-hydroxyphenylglycolaldehyde	0.56	
			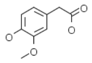 Homovanillate	0.56	
			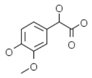 Vanillylmandelic acid	0.53	
17	**wogonin**	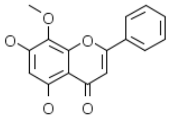	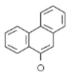 9-Hydroxyphenanthrene	0.71	transient receptor potential melastatin 4

			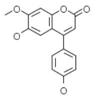<br>Melannin	0.62	
18	kavain	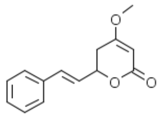	N/A		
19	dihydrokavain	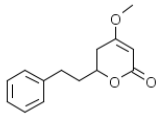	N/A		
20	**indirubin**	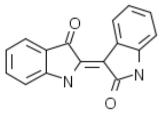	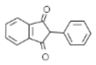<br>Phenindione	0.76	prothrombin
			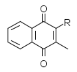<br>Vitamin K	0.62	

Supplementary Table S3. MAOB binding parameters determined by ITC.

Compounds	K_d/μM	ΔH/Kcal mol^{-1}	ΔS/cal mol^{-1} degree^{-1}
synephrine	183±10.3	-3.9±1.4	-41.8
danshensu	16±0.8	-15.1±0.8	-18.7
vanillin	165±9.1	-2.3±0.8	-52.7
selegiline	15±2.4	-18.4±2.2	-33.2

Supplementary Table S4. Primers for determining levels of gene expression.

gene name	Forward primer	Reverse primer
MAOB	5′-ATA TGG AAG GGT TCT ACG CC-3′	5′-AAA CTG GTG AAA CAG AAC GC-3′
BIRC2	5′-CCA GCC TGC CCT CAA ACC CTC T-3′	5′-GGG TCA TCT CCG GGT TCC CAA C-3′
BIRC3	5′-ACA TTT CCC CAG CTG CCC ATT C-3′	5′-CTC CTG CTC CGT CTG CTC CTC T-3′
BIRC5	5′-CTG ATT TGG CCC AGT GTT TT-3′	5′-TCA TCT GAC GTC CAG TTT CG-3′
CXCR4	5′-AGC ATG ACG GAC AAG TAC C-3′	5′-GAT GAT ATG GAC AGC CTT ACA C-3′
IL1B	5′-GAG AAC CAA GCA ACG ACA AAA TAC C-3′	5′-GCA TTA GAA ACA GTC CAG CCC ATA C-3′
IL6	5′-GAC AAC TTT GGC ATT GTG G-3′	5′-ATG CAG GGA TGA TGT TCT G-3′
CDH1	5′-GGA TTG CAA ATT CCT GCC ATT C-3′	5′-AAC GTT GTC CCG GGT GTC A-3′
VIM	5′-GAC AAT GCG TCT CTG GCA CGT CTT-3′	5′-TCC TCC GCC TCC TGC AGG TTC TT-3′
FN1	5′-TGA CCT TTT CTG GCT CGT CT-3′	5′-GTT CAG CAC AAA GGG CTC TC-3′

Supplementary Table S5. Primers used for ChIP assays.

gene name	Forward primer	Reverse primer
CXCL8	5′-GGG CCA TCA GTT GCA AAT C-3′	5′-TTC CTT CCG GTG GTT TCT TC-3′
NFKBIA	5′-GAC GAC CCC AAT TCA AAT CG-3′	5′-TCA GCT CGG GGA ATT TCC-3′
CXCR4	5′-ACA GAG AGA CGC GTT CCT AG-3′	5′-AGC CCA GGG GAC CCT GCT G-3′
IL1B	5′-TCC CTG GAA ATC AAG GGG TGG-3′	5′-TCT GGG TGT GCA TCT ACG TGC C-3′
IL6	5′-AAG CAC ACT TTC CCC TTC C-3′	5′-CTA TCG TTC TTG GTG GGC TC-3′
ACTB	5′-TGC ACT GTG CGG CGA AGC-3′	5′-TCG AGC CAT AAA AGG CAA-3′

Supplementary Methods

Chemicals, antibodies, and reagents. Danshensu, selegiline, pyrrolidine dithiocarbamate, and TG101209 were obtained from Sigma (St. Louis, MO) or EMD-Calbiochem (La Jolla, CA). Antibodies specific for MAOB, Tubulin, pIκBα, p65, Lamin A/C, cIAP1, cIAP2, Survivin, CX-C chemokine receptor type 4, interleukin (IL)-1β, IL-6, E-cadherin, vimentin, and fibronectin were purchased from Santa Cruz Biotechnology (Santa Cruz, CA), Abcam (AbCAM, Cambridge, UK) or Cell Signaling Technology (Beverly, MA) for Western blot analysis. Cell culture medium (RPMI-1640), FBS, glutamine, penicillin, streptomycin, and Trizol® were obtained from Sigma or Gibco (Grand Island, NY). Control siRNA and MAOB-specific siRNA were purchased from Dharmacon (Lafayette, CO).

Real-time quantitative RT-PCR (qRT-PCR). Briefly, aliquots of a master mix containing all reaction components and primers were added to a real time PCR plate (Applied Biosystems, Foster City, CA). All PCR reagents were supplied with SYBR Green core reagent kits (Applied Biosystems). The primers used for qRT-PCR are described in Supplementary Table S4. *MAOB* gene expression was measured in triplicate. qRT-PCR was performed using an Applied Biosystems-7900 HT qRT-PCR instrument using 40 cycles of 15 s at 95℃ and 1 min at 60℃ followed by thermal denaturation. Gene expressions versus *GAPDH* mRNA were determined using the $2^{-\Delta\Delta C_T}$ method[1]. To simplify data presentation, relative expression values were multiplied by 10^2.

Western blot analysis and transient transfection. Whole cell lysates were extracted using RIPA lysis buffer (50 mM Tris, pH 7.4, 150 mM NaCl, 1% Triton X-100, 25 mM NaF, 1 mM DTT, 20 mM EGTA, 1 mM Na_3VO_4, 0.3 mM PMSF, and 5 U/mL Aprotinin) and lysate protein concentrations were determined using the Bio-Rad protein assay kit (Bio-Rad Laboratories, Hercules, CA). To obtain the cytoplasmic extract (CE), cells were suspended in buffer A (10 mM HEPES, pH 7.9, 50 mM NaCl, 1 mM DTT, 0.1 mM EDTA, 1 mM PMSF, 1 μg/mL Aprotinin, 5 μg/mL Leupeptin, and 1 μg/mL Pepstatin A) and then incubated for 20 min on ice. An equal volume of buffer B (buffer A + 0.1% NP-40) was then added and cells were placed on

ice for 20 min. Samples were then centrifuged at 5,000 g for 2 min at 4°C to remove cellular debris and the CE was collected. Samples were then separated by SDS-PAGE, transferred to nitrocellulose membranes, which were blocked with 5% skim milk in TBS containing Tween 20 (10 mM Tris, 100 mM NaCl, and 0.1% Tween 20) at room temperature (RT) for 30 min. Membranes were subsequently probed with specific primary antibodies and peroxidase-conjugated secondary antibody (Santa Cruz Biotechnology, Santa Cruz, CA) and its binding was visualized using an enhanced chemiluminescence detection system (Roche Applied Science, Penzberg, Germany). For transient transfection, cells were plated at a density of 5×10^5 cells/well in 6-well dishes and then incubated for 4 h. Cells were then transiently transfected with siRNA oligonucleotides (10 nM) using DharmaFECT 1 (Dharmacon, Lafayette, CO) according to the manufacturers' instructions.

Molecular modeling. In order to determine the binding affinities of an inhibitor and its analogs, an *in silico* substrate docking experiment was performed using AutoDock Vina[2]. A known MAOB inhibitor (selegiline) and three structural analogs (danshensu, synephrine, and vanillin) were geometrically optimized using a semi-empirical quantum mechanical method (AM1) and electronic Ligand Builder and Optimization Workbench (eLBOW)[3], prior to modeling docking. Optimized substrate and analogs were docked into the Protein Data Bank (PDB) deposited crystallographic structure of MAOB (2VRM). Stochastic global optimization scoring function was used to search for the active-site of MAOB. After confirming that the located active-site and the previously identified site agreed, elaborated modeling was performed. Multiple (> 5,000) steps of conformation perturbation were followed by local optimization using the Broyden-Fletcher-Goldfarb-Shanno algorithm.

Structural comparison. In order to select human metabolite mimetic NF-κB inhibitors, a large-scale structural comparison of selected TOM-derived compounds and human metabolites was performed. Structures of selected TOM-derived compounds were systematically compared with those of human metabolites in a pairwise manner. For this, electronic files (CDX, SDF, or MOL) containing structural

information of the selected compounds and human metabolites were downloaded from the TCM Database@Taiwan2 and from the Kyoto Encyclopedia of Genes and Genomes, respectively[4,5]. Structural comparisons were conducted using the Small Molecule Subgraph Detector (SMSD) using its default settings (bond sensitive, remove hydrogens, and stereo filter)[6]. This procedure was automated to provide pairwise comparisons between selected TOM and human metabolites. TOM compounds were considered to be structurally similar to human metabolites if they had similarity scores of ≥0.5 and ≤1.0 on the 0 to 1 SMSD scale.

Isothermal titration calorimetry. Recombinant human MAOB was purchased from Sigma. Direct interactions between MAOB and selected compounds were measured using a MicroCal VP-ITC instrument (GE Healthcare, Pittsburgh, PA) at 25°C using a standard procedure. Recombinant MAOB was equilibrated with 20 mM Tris buffer (pH 7.5) containing 150 mM NaCl. Protein was added to the calorimetric reaction cell at 0.01 mM and titrated with 0.1 mM compound in the same buffer. Protein and ligand solutions were degassed prior to use. Each titration experiment was performed with 29 injections of 10 μL at 300-s equilibration intervals. Heats of dilution for ligands were determined by titrating the ligand in the same buffer without protein and used to correct protein titrations. Data were fitted to a single site binding model by non-linear least-squares regression using the Origin software package. Data fits yielded binding affinities (K_d), enthalpy changes (ΔH), entropy changes (ΔS), and binding stoichiometries for the titration.

Thiazolyl blue tetrazolium bromide assay (cell viability assay). NSCLC cells were seeded and cultured in a 24-well plate with or without danshensu at different conditions for 4 h. Media were then removed, 0.05% thiazolyl blue tetrazolium bromide solution (Sigma) was added, and cells were incubated at 37°C for 2 h. The thiazolyl blue tetrazolium bromide solution was then replaced with dimethyl sulfoxide and plates were incubated for 10 min. After incubation, solutions were transferred to a 96-well plate in duplicate and absorbances were measured.

MAOB activity. MAOB activities were determined using the Amplex Red Monoamine Oxidase Assay kit (Molecular Probes Inc., Eugene, Oregon) as previously described[7] with several modifications. Briefly, 5 μg of recombinant human MAOB (Sigma, St. Louis, MO) was incubated with 16 μM of the MAOB substrate benzylamine with or without danshensu or selegiline for 30 min (drugs and substrate were dissolved in deionized water). Activities were assessed by measuring amounts of H_2O_2 generated with 10-acetyl-3,7-dihdroxyphenoxazine in a horseradish peroxidase-coupled reaction. Fluorescences were measured using a multiplate reader (CytoFluor 4000, PerSeptive Biosystems, Framingham, MA) at excitation/emission wavelengths of 30/580 nm, respectively. The log(inhibitor) vs. response equation of nonlinear regression parameters in GraphPad Prism Software (La Jolla, CA) was used to determine IC_{50} values for MAOB inhibition.

ChIP assay. After transfection, irradiation, and/or drug treatments, NSCLC cells (5×10^8) were cross-linked with 1% formaldehyde, quenched in 125 mM glycine, washed with PBS, lysed and sonicated. Extracts were centrifuged, diluted in dilution buffer (0.01% SDS, 1.1% Triton X-100, 1 mM EDTA, 20 mM Tris-HCl, pH 8.1, and 200 mM NaCl), and precleared with protein A/G-agarose and calf thymus DNA at 4 °C for 1 h. Next, immunoprecipitation was performed using anti-p65 or -IgG antibody. Immunoprecipitates were collected and washed in low salt buffer (0.1% SDS, 1% Triton X-100, 2mM EDTA, 20 mM Tris-HCl, pH 8.1, and 150 mM NaCl), high salt buffer (same contents as low salt buffer but containing 500 mM NaCl), and LiCl wash buffer (0.25 M LiCl, 1% Nonidet P-40, 1% deoxycholate, 1 mM EDTA, and 10 mM Tris-HCl, pH 8.1). They were then washed twice in 10 mM Tris, 5mM EDTA. DNA was extracted from beads using 100 μL of elution buffer (1% SDS and 0.1 M NaHCO) supplemented with 0.25 M NaCl. Following overnight incubation at 65°C (to break cross-links), samples were incubated for an additional 1 h at 65°C with 10 μM EDTA, 40 μM Tris, pH 6.8, and 2 μg of proteinase K. DNA was purified using the QIAquick PCR purification kit (Qiagen, Valencia, CA). PCR was performed using primers encompassing the human *CXCL8*, *NFKBIA*, *CXCR4*, *IL1B*, *IL6* and *ACTB* promoters. The primers used are described in Supplementary Table S5.

DNA fragmentation assay. Cells were seeded at a density of 4 × 10^5 cells in 96-well plates, incubated overnight, and then exposed to IR, drugs and/or siRNA. Apoptosis induction was determined by analyzing cytoplasmic histone-associated DNA fragmentation using a cell death detection kit (Roche Applied Science, Mannheim, Germany).

Colony forming assay. Cells were plated at a density of 300 cells in 6-well dishes. After treatment with IR, drugs, and/or siRNA, cells were subsequently grown for 14 days, then fixed with 10% methanol/10% acetic acid and stained with 1% crystal violet. Colonies containing more than 50 cells were counted using densitometric software and scored as survivors[8].

Concentration of media and Coomassie blue staining. A549 or NCI-H1299 cells were grown to confluence in RPMI containing 10% FBS. After treatment, media were collected and centrifuged at 2,000 g and 4°C for 10 min. Supernatants were concentrated 5-fold using Amicon Ultracell 10K filters (Millipore, Billerica, MA) and proteins in the concentrated media were verified by SDS-PAGE followed by Western blot analysis, which was performed as described above. To confirm equal loadings, sample-loaded gels were stained using 0.25% Coomassie brilliant blue R-250 (Sigma), and then destained in methanol: acetic acid: distilled water (3:1:6).

Cytokine-specific enzyme-linked immunosorbent assay (ELISA). Cells (8 × 10^5) were plated in 6-well plates and grown to 80% confluence. After treatments, amounts of IL-1β and IL-6 released into culture media were determined using ELISA kits (R&D Systems, Abingdon, UK).

Cell assay (3D culture) and immunofluorescence (IF) staining. Matrigel (BD Biosciences, Franklin Lakes, NJ) was thawed overnight at 4°C and mixed well using prechilled pipette tips before use. Cells were cultured on eight-well chambered glass slides (Nunc, Naperville, IL) and they were incubated at 37°C for 30 min. Cells were harvested, counted, and resuspended as a suspension of 25,000 cells/mL in RPMI medium, and 200 μL of this suspension was then mixed with 200 μL of RPMI medium containing 4% Matrigel and

this mixture was dispensed into each well of the glass slides, and incubated for 3 d. For drug treatment and irradiation, medium was exchanged for 400 μL of RPMI containing 4% Matrigel and 50 μM danshensu, 2 μM selegeline or 20 μM TG101209. Cells were further incubated for 4 h, exposed to IR, and 24 h later, cells and acini were analyzed by IF staining. Briefly, cells and acini were fixed with 2% paraformaldehyde for 20 min, permeabilized in 0.5% Triton X-100 for 10 min, washed three times with PBS and blocked in IF buffer (PBS, 0.1% BSA, 0.2% Triton X-100, and 0.05% Tween-20) containing 10 % FBS at 37℃ for 30 min. They were then incubated with primary antibody against Tubulin overnight at RT and washed three times with IF buffer. After probing with DyLight 488-conjugated secondary antibodies (Thermo Scientific) and counterstaining with 4′,6-diamidino-2-phenylindole, slides were mounted with VECTASHIELD Hard-Set Mounting Medium (Vector Laboratories, Burlingame, CA). Fluorescent images were obtained using an Olympus IX71 fluorescence microscope (Olympus Optical Co. Ltd., Tokyo, Japan).

Transwell cell migration assay. To evaluate the migration capacity of NSCLC cells, a transwell cell migration assay was conducted as previously described[9]. Cell migration assays were performed in a 24-well Transwell chamber (Corning, Inc., Corning, NY). Cells (1×10^4 in serum-free RPMI-1640) cultured with or without desired treatments for 72 h were seeded in the upper chambers which were each equipped with a 5-μm pore insert. Lower chambers were filled with 600 μL of RPMI-1640 containing 2% FBS. Upper membrane surfaces were wiped with a cotton swab 6 h later to remove cells that had not migrated into the lower chamber. Cells that had migrated and attached to the lower membrane surface were fixed with 4% paraformaldehyde and hematoxylin stained for counting. Migration was normalized versus the number of untreated cells that had migrated.

Wound healing assay. Monolayers of cells that had reached 80% confluency in RPMI-1640 medium supplemented with 1% FBS were scratched using a 200 μL pipette tip. Cells were then incubated with fresh medium with or without danshensu, selegiline, or TG101209 for 17 h. Photomicrographs were taken at 100× using an Olympus IX71 fluorescence microscope (Olympus Optical Co. Ltd.).

Animal protocol and tumor xenograft in nude mice. Six-week-old male BALB/c athymic nude mice (Central Lab Animals Inc., Seoul) were used for the *in vivo* experiments. The protocols used were approved by the Institutional Animal Care and Use Committee of Pusan National University (Busan, Republic of Korea) and were in accord with the provisions of the NIH Guide for the Care and Use of Laboratory Animals. Mice were housed individually or in groups of up to five mice in sterile cages in an animal care facility at 23±1˚C under a 12 h light/dark cycle and quarantined for 1 wk prior to the study. Animals were fed water and a standard mouse chow diet *ad libitum*. Initially, they were injected with 2×10^6 A549 or NCI-H1299 cells in a flank and tumors were allowed to develop. Upon identification of a palpable tumor (minimum volume 200 mm^3), DMSO or danshensu (200 μg/kg body weight) were administered intraperitoneally every day for 30 d. Animals were also irradiated with 10 Gy once a week for 4 wk from the day of treatment commencement. Tumor lengths (L) and widths (l), which were measured using a caliper, were used to calcuate tumor volumes using $(L \times l^2)/2$. At the end of the treatment period, animals were sacrificed and tumor tissues were Western blotted.

Supplementary References

1. Livak, K. J. & Schmittgen, T. D. Analysis of relative gene expression data using real-time quantitative PCR and the 2(-Delta Delta C(T)) Method. *Methods* **25,** 402-408 (2001).
2. Trott, O. & Olson, A. J. AutoDock Vina: improving the speed and accuracy of docking with a new scoring function, efficient optimization, and multithreading. *J. Comput. Chem.* **31,** 455-461 (2010).
3. Moriarty, N. W., Grosse-Kunstleve, R. W. & Adams, P. D. electronic Ligand Builder and Optimization Workbench (eLBOW): a tool for ligand coordinate and restraint generation. *Acta Crystallogr. D Biol. Crystallogr.* **65,** 1074-1080 (2009).
4. Chen, C. Y. TCM Database@Taiwan: the world's largest traditional Chinese medicine database for drug screening in silico. *PLoS One* **6,** e15939 (2011).
5. Kanehisa, M. *et al.* Data, information, knowledge and principle: back to metabolism in KEGG. *Nucleic Acids Res.* **42,** D199-205 (2014).
6. Rahman, S. A., Bashton, M., Holliday, G. L., Schrader, R. & Thornton, J. M. Small Molecule Subgraph Detector (SMSD) toolkit. *J. Cheminform.* **1,** 12 (2009).
7. Rocha, M. A., Crockett, D. P., Wong, L. Y., Richardson, J. R. & Sonsalla, P. K. Na(+)/H(+) exchanger inhibition modifies dopamine neurotransmission during normal and metabolic stress conditions. *J. Neurochem.* **106,** 231-243 (2008).
8. Kim, W., Youn, H., Kang, C. & Youn, B. Inflammation-induced radioresistance is mediated by ROS-dependent inactivation of protein phosphatase 1 in non-small cell lung cancer cells. *Apoptosis* **20,** 1242-1252 (2015).
9. Kang, J. *et al.* Rhamnetin and cirsiliol induce radiosensitization and inhibition of epithelial-mesenchymal transition (EMT) by miR-34a-mediated suppression of Notch-1 expression in non-small cell lung cancer cell lines. *J. Biol. Chem.* **288,** 27343-27357 (2013).
